# Supplementary material for: Closed-loop brain stimulation augments fear extinction in male rats
Source: Nat Commun. 2023 Jul 5;14:3972. doi: 10.1038/s41467-023-39546-7 (PMC10322911; doi:10.1038/s41467-023-39546-7)
Supplement: Supplementary file 1 — Supplementary Information [file 41467_2023_39546_MOESM1_ESM.pdf]

Supplementary Information

Title: **Closed-loop brain stimulation augments fear extinction in male rats**

Author names: Rodrigo Ordoñez Sierra[1†], Lizeth Katherine Pedraza[1†], Lívia Barcsai[1,2,3], Andrea Pejin[1,2,3], Qun Li[1], Gábor Kozák[1], Yuichi Takeuchi[1,4], Anett J. Nagy[1,2,3], Magor L. Lőrincz[1,5,6], Orrin Devinsky[7], György Buzsáki[8,9], Antal Berényi[1,2,3,8*]

[†] These authors contributed equally to this work.

Author affiliations:

[1]MTA-SZTE 'Momentum' Oscillatory Neuronal Networks Research Group, Department of Physiology, University of Szeged, Szeged, 6720, Hungary.

[2]HCEMM-SZTE Magnetotherapeutics Research Group, University of Szeged; Szeged, 6720, Hungary

[3]Neunos Inc; Boston, MA 02108, USA

[4]Department of Biopharmaceutical Sciences and Pharmacy, Faculty of Pharmaceutical Sciences, Hokkaido University, Sapporo, Japan

[5]Department of Physiology, Anatomy and Neuroscience, Faculty of Sciences University of Szeged; Szeged, 6726, Hungary

[6]Neuroscience Division, Cardiff University, Museum Avenue, Cardiff CF10 3AX, UK

[7]Department of Neurology, NYU Langone Comprehensive Epilepsy Center, NYU Grossman School of Medicine, New York, NY 10016, USA

[8]Neuroscience Institute, New York University; New York, NY 10016, USA

[9]Center for Neural Science, New York University, New York, NY 10016, USA

[*]**Corresponding author:** Antal Berényi (drberenyi@gmail.com)

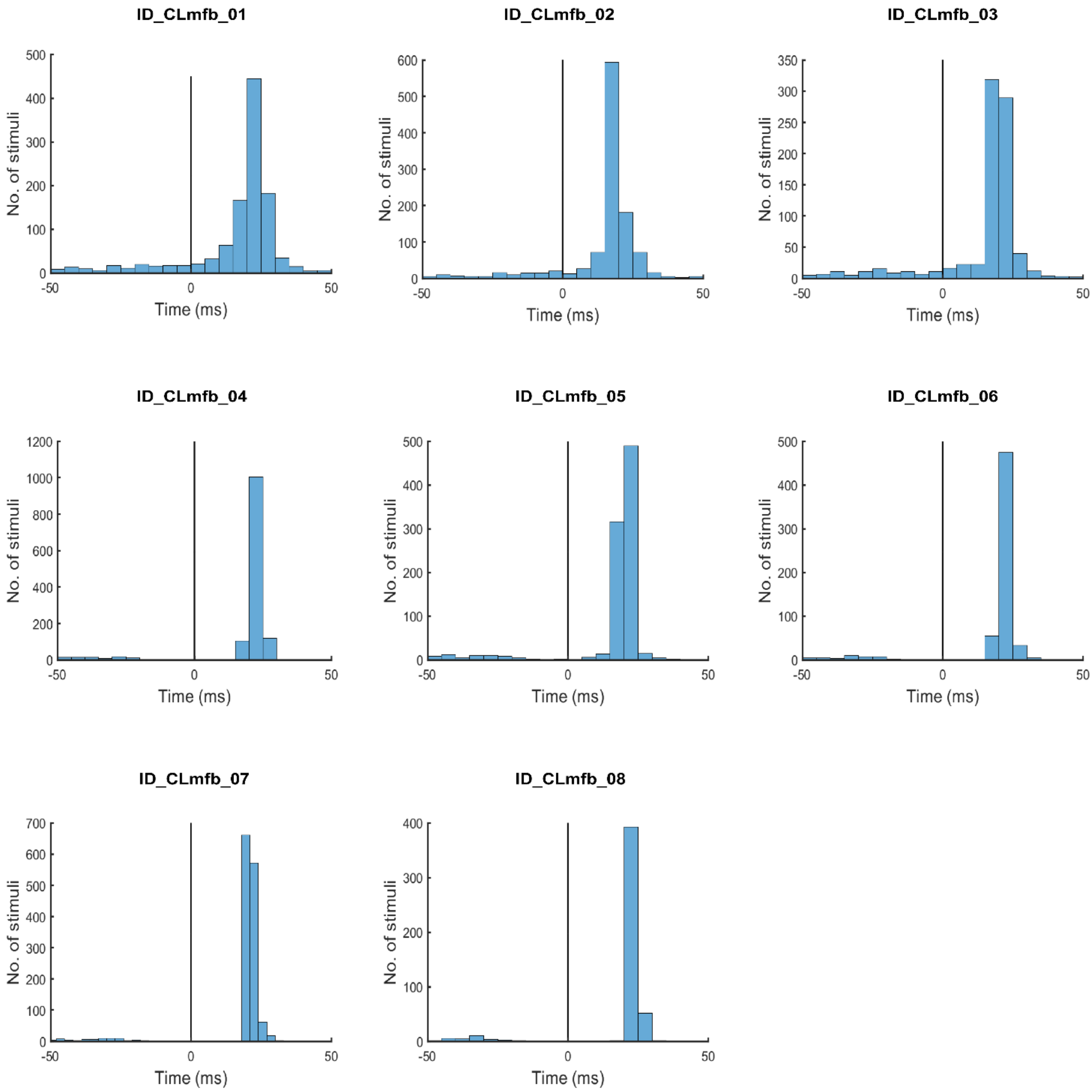

Supplementary Fig. 1. Cross-correlograms for stimulation onset in closed-loop animals. Individual cross-correlograms (n = 8 from Fig. 1) of stimulations and offline-detected SWRs in closed-loop animals. The minimum delay for trigger the stimulation after SWR detection was 15ms and maximum 27 ms. The largest number of stimuli (blue peak) were delivered between 18-21 ms after the SWR onset (black line: time zero).

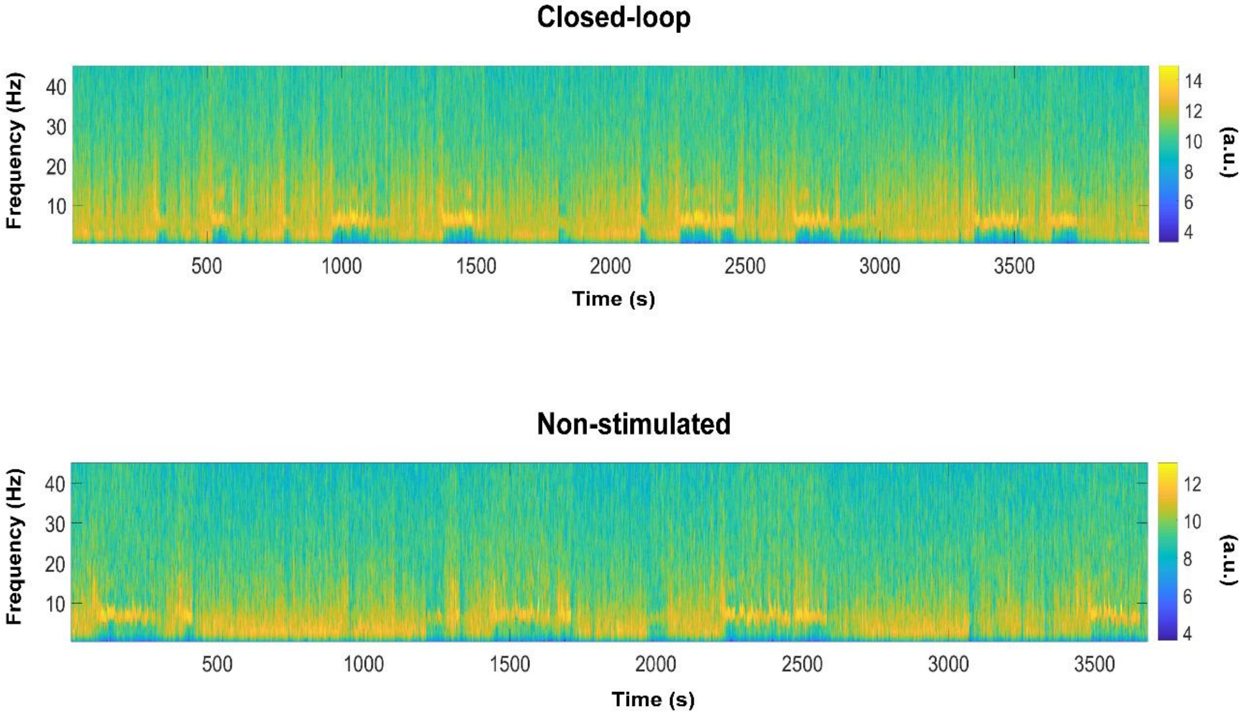

Supplementary Fig. 2. Architecture of sleep during stimulation. Examples of power spectrograms (log scale) of hippocampal local field potentials recorded during a sleep session in stimulated (closed-loop) and non-stimulated animals.

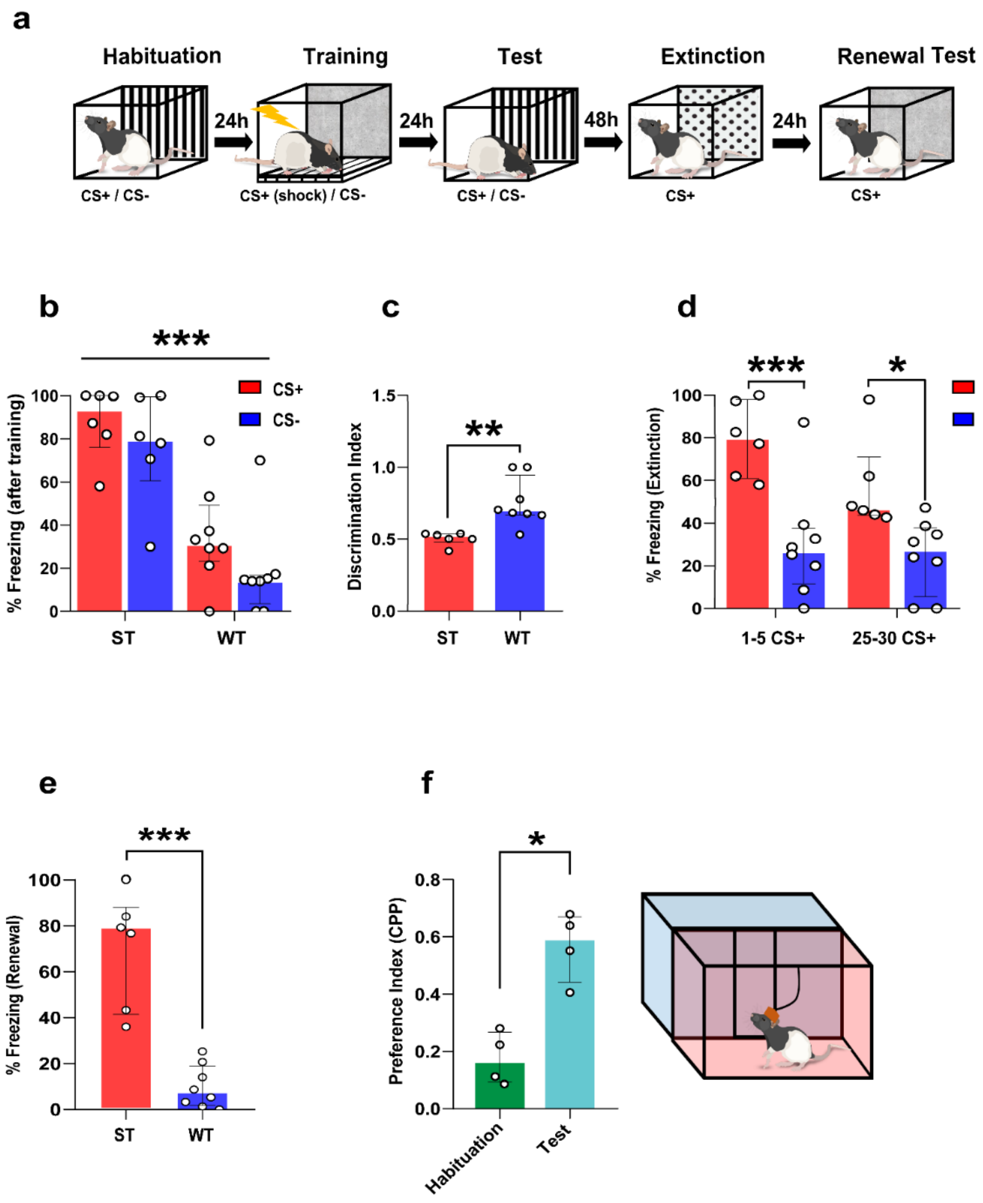

Supplementary Fig. 3. Strong fear conditioning induces a PTSD-like phenotype. a Schematics of the experimental design. **b** Animals submitted to strong training (ST; n = 6) during fear conditioning (5 × 1 mA) showed robust fear reaction compared to a weak training (WT; n = 8) (5 × 0.5 mA) either CS+ and CS- test. However, there is no significant differences within the same group regarding fear reactions to CS+ or CS- (mixed ANOVA: $F(1,12) = 22.68$, $P < 0.0005$, Tone factor (CS+ vs CS-). **c** Fear generalization analysis using a discrimination index showed that strong training induces poor discrimination between CS+ and CS- compared to a weak training (Mann Whitney test: $U = 2$, $P =$

0.0027, two-tailed). **d** During extinction, animals in the strong training group expressed high fear responses during the initial and last 5 blocks of CS+ compared to the weak training. Moreover, there is no significant decrease of freezing levels in both groups across the extinction (0-5 min to 25-30 min) (mixed ANOVA: $F(1,10) = 25.35$, $P < 0.0005$, group factor). **e** Animals exposed to renewal test in a hybrid context and submitted to strong fear conditioning expresses high fear reactions compared to animals trained with a weak training (Mann Whitney test: $U = 0$, $P = 0.0007$, two-tailed). **f** In order to assess the rewarding properties of the MFB stimulation, animals (n = 4) were submitted to conditioning place preference (see Material and Methods). Animals expressed a significant preference for the chamber paired with the MFB stimulation during the test compared to the habituation session (Mann Whitney test: $U = 0$, $P = 0.0286$, two-tailed). * $P < 0.05$, ** $P < 0.01$, *** $P < 0.001$. Bar plots and error bars represent medians and interquartile ranges, individual data points are also displayed. Detailed statistics are shown in Supplementary Data 1. Source data provided as a Source Data file. Silhouettes on panels **a** and **f** are obtained from https://github.com/eackermann/ratpack under MIT License.

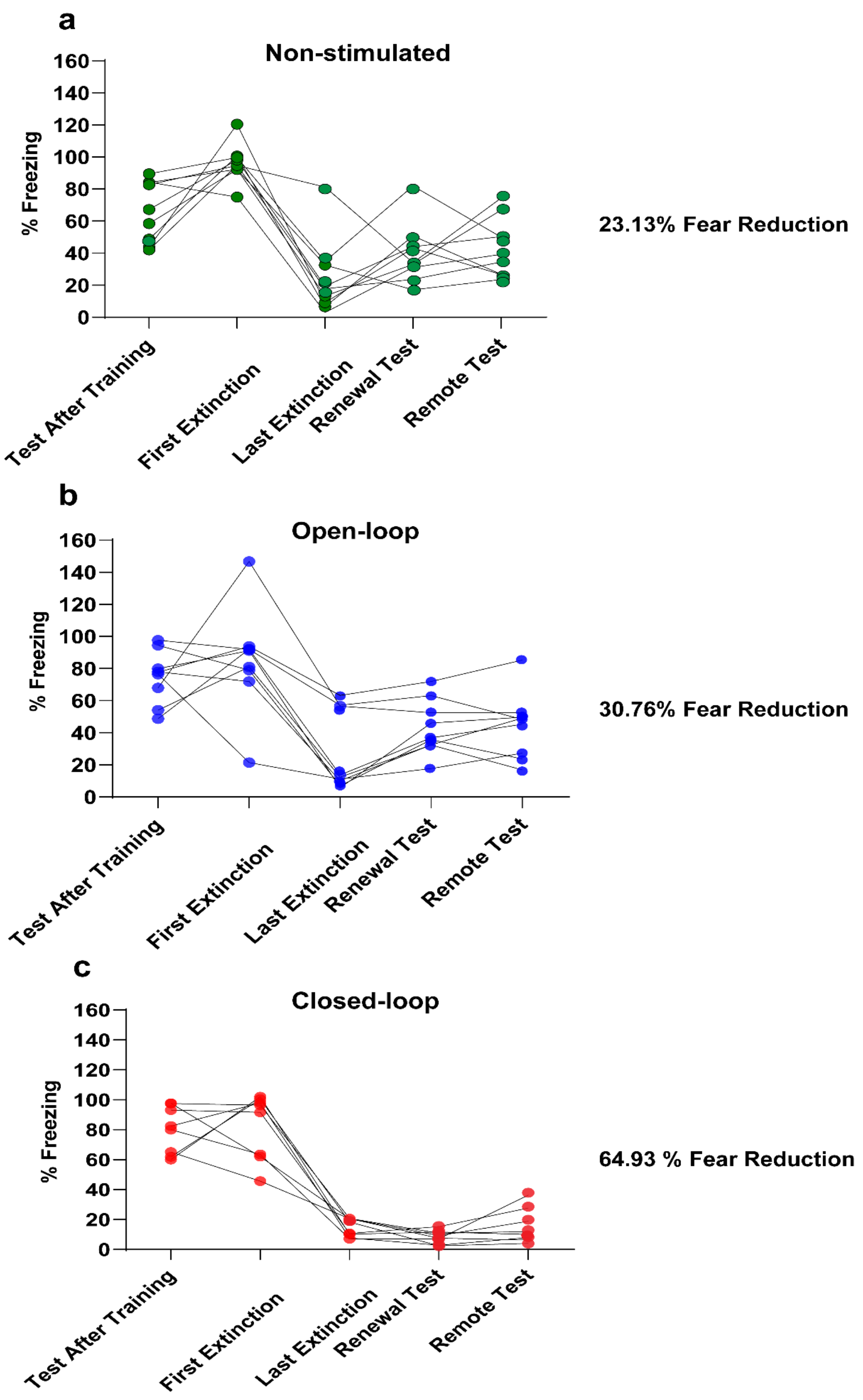

Supplementary Fig. 4. Individual fear expression across sessions. Graphs display individual performances in **a** non-stimulated, **b** open-loop and **c** closed-loop experiments. Percentages (right to each graph) show fear reduction from a Δ freezing analysis (see Fig. 1) comparing test to CS+ and remote test (25 days after extinction). Non-stimulated (23.13%, n = 9), Open-Loop (30.76%, n = 9), Closed-loop (64.93%, n = 8). Source data provided as a Source Data file.

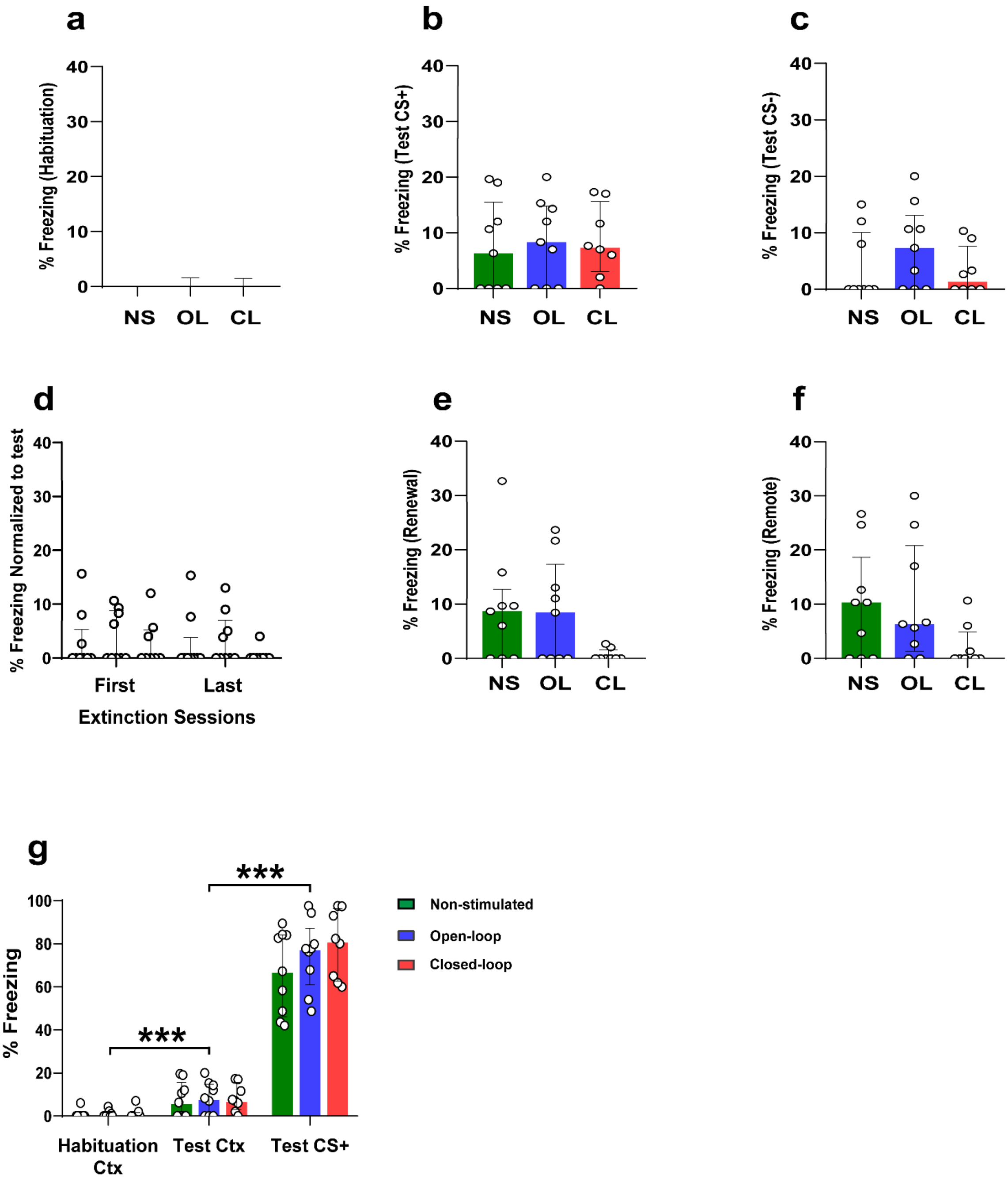

Supplementary Fig. 5. Contextual fear conditioning across sessions. No differences were detected across session in fear expression before CS+ onset between groups across sessions (non-stimulated (NS) n = 9; open-loop (OL) n = 9; closed-loop (CL) n = 8): **a** Habituation (Ctx) (Kruskal-Wallis test: H = 0.8939, P = 0.6396), **b** Test CS+ (Kruskal-Wallis test: H = 0.2427, P = 0.8857), **c** Test CS- (Kruskal-Wallis test: H = 2.247, P = 0.3251), **d** Extinction (mixed ANOVA: F (2,23) = 0.4253, P = 0.6586, group x time interaction), **e** Renewal (Kruskal-Wallis test: H = 4.867, P = 0.0877) and **f** Reinstatement (Kruskal-Wallis test: H = 3.927, P = 0.1404). **g** Compared to habituation, a significant

fear increase was detected in all groups before CS+ onset during test. This increase was significantly lower compared with fear expression during CS+ presentations (5 CS+ average) (mixed ANOVA: $F_{(1.203, 41.5123)} = 354.3$, $P < 0.0001$, time factor). *** $P < 0.001$. Bar plots and error bars represent medians and interquartile ranges, individual data points are also displayed. Detailed statistics are shown in Supplementary Data 1. Source data provided as a Source Data file.

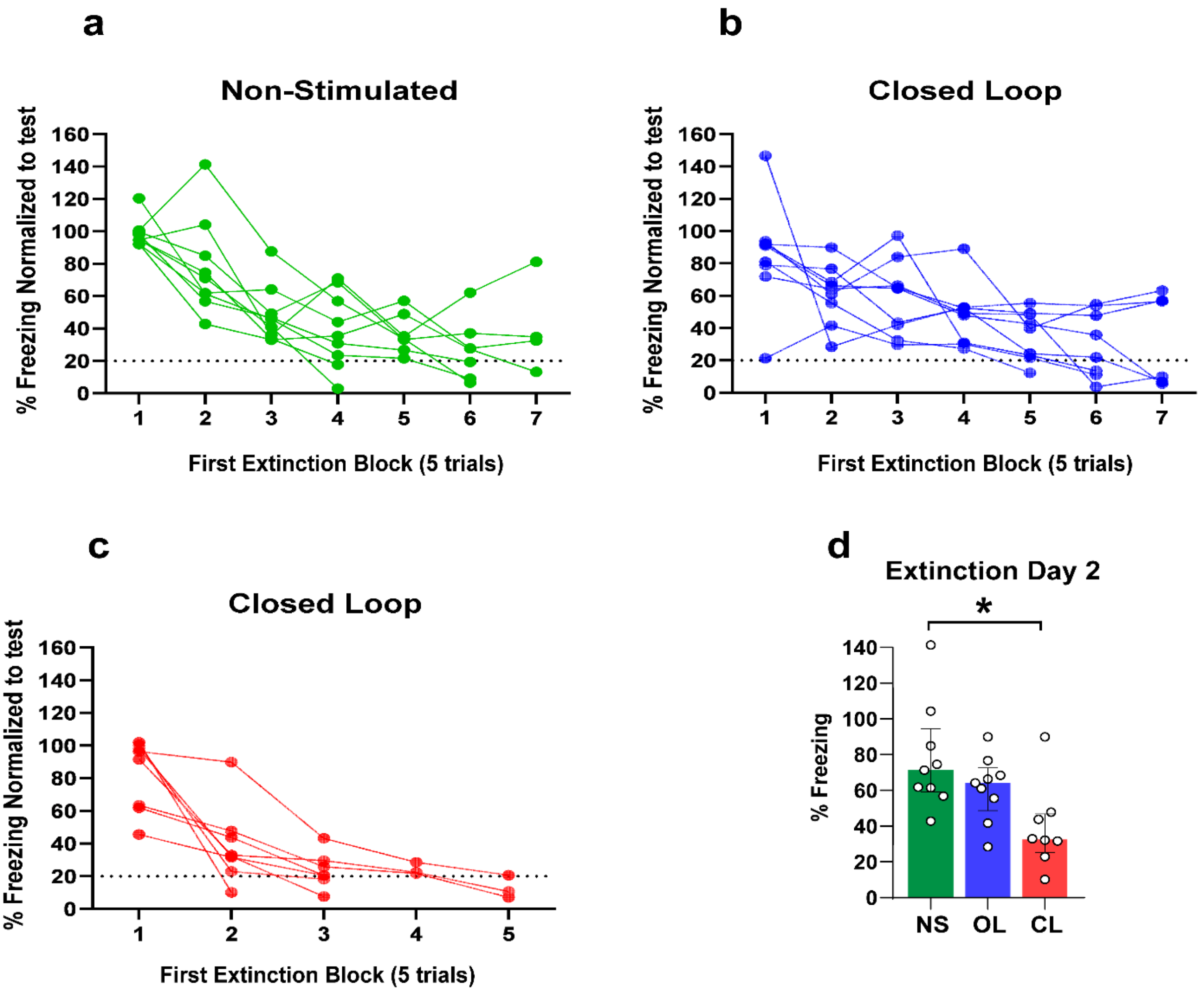

Supplementary Fig. 6. Individual extinction performance across sessions. All groups were showing significant reduction of fear expression across the extinction sessions (Fig. 1h). Individual performance was assessed during the first extinction block every day (average of first 5 CS+ presentations) as indicative of remission **a-c** (see Material and Methods). **d** Even with only 1 intervention, significant differences between non-stimulated and closed-loop animas were detected in the extinction session two (Kruskal-Wallis test: H = 7.909, P = 0.0192, non-stimulated (NS) n = 9; open-loop (OL) n = 9; closed-loop (CL) n=8). * P < 0.05. Bar plots and error bars represent medians and interquartile ranges, individual data points are also displayed. Detailed statistics are shown in Supplementary Data 1. Source data provided as a Source Data file.

ACC
+1.60mm

VHC
-1.40 mm

MFB
-2.56 mm

AMY
-3.14 mm

dHPC
-3.60 mm

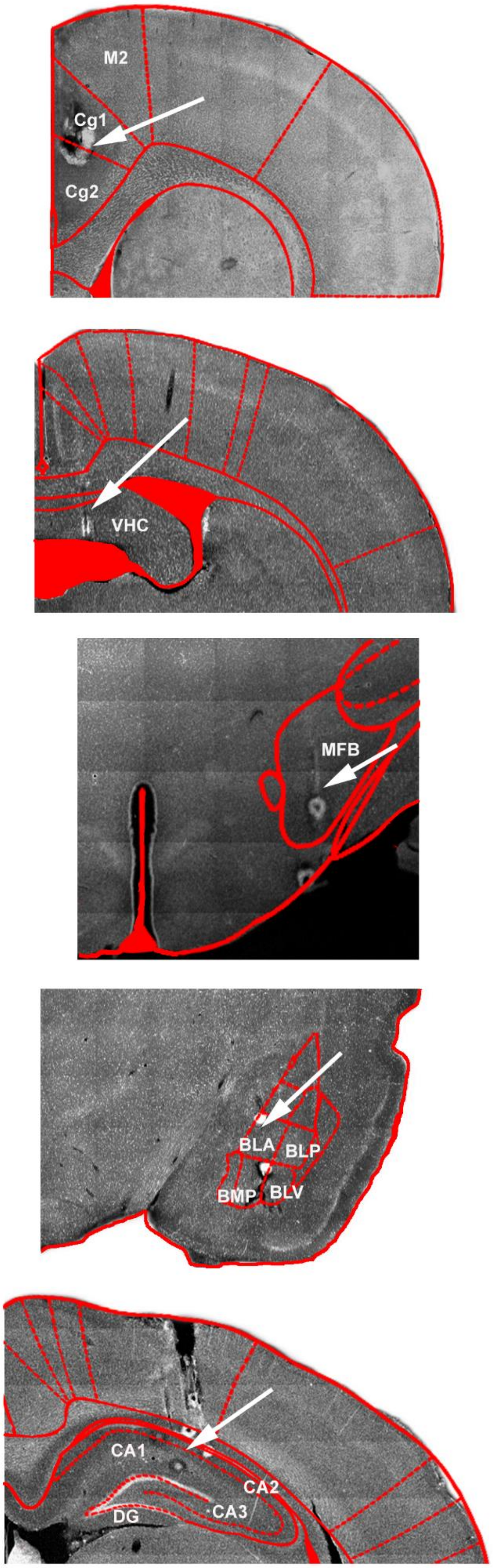

Supplementary Fig. 7. Histological verification of electrode placements. A coronal section of the anterior cingulate cortex (ACC), ventral hippocampal commissure (VHC), medial forebrain bundle (MFB), Amygdala (AMY) and dorsal hippocampus (dHPC) stained with DAPI is shown. Recording sites on each shank were lesioned (white dots) after the end of the experiment by applying 100 μA anodal direct current for 10 s via electrode tips. White arrows point to the histological traces of representative electrode trajectories. The position of the electrodes was maintained throughout all experiments requiring neuronal recording or stimulation. (Fig. 1-4, Supplementary Fig. 8). Abbreviations: M2: secondary motor cortex; Cg1&2: cingulate cortex; VHC: ventral hippocampal commissure; MFB: medial forebrain bundle; BLA, BLP, BLV: anterior, posterior and ventral nuclei of basolareal amygdala; BMP: nucl. posterior of basomedial amygdala; CA1-2-3: Cornu Ammonis 1-2-3 fields of hippocampus; DG: dentate gyrus.

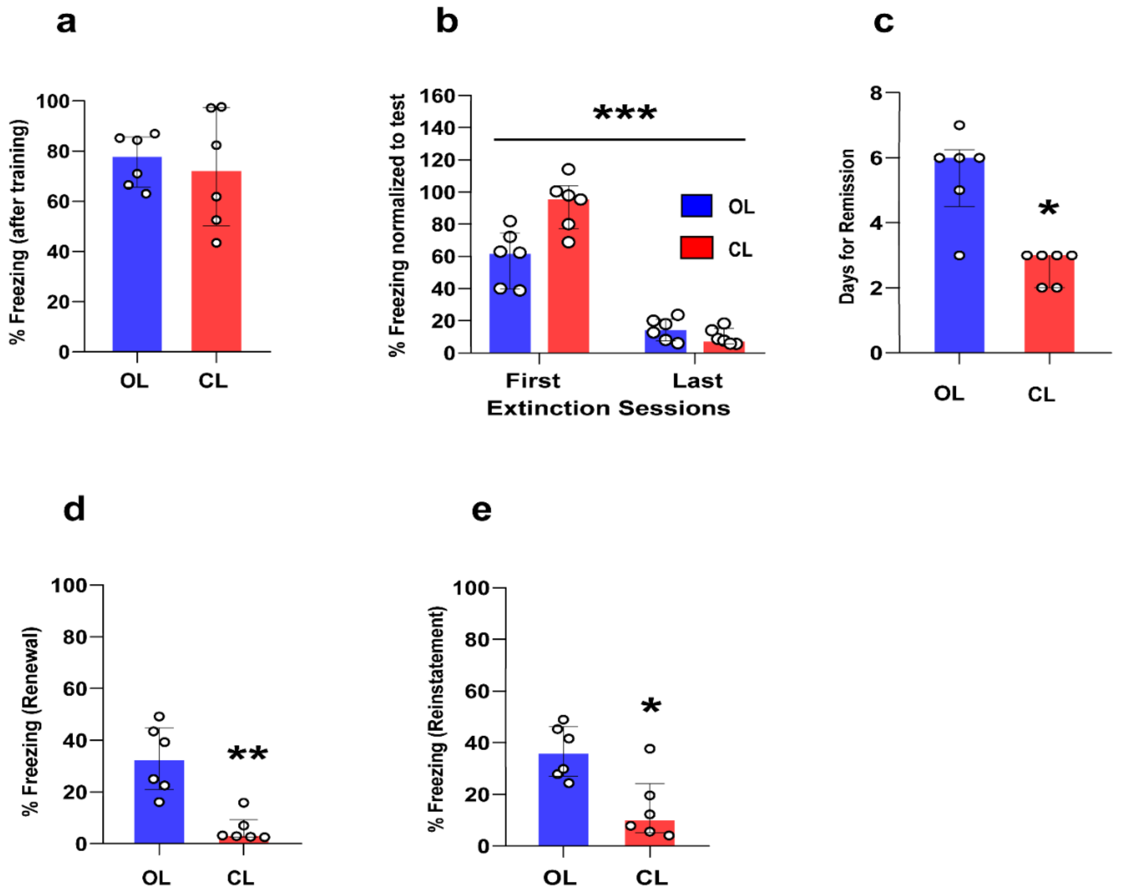

Supplementary Fig. 8. Extinction enhancement induced by closed-loop neuromodulation is not affected by previously consolidated memories. Data from animals submitted to concomitant T-maze task and closed-loop neuromodulation for extinction enhancement. **a** During the test to the CS+, there are no significant differences between the groups showing that all groups are able to acquire the fear conditioning (Mann Whitney test: U = 15, P = 0.6991, two-tailed). Open-loop (OL) n = 6, Closed-loop (CL) n = 6. **b** No differences were found in the fear expression between groups during the first 5 CS+ block from first and last extinction day. There was a significant decrease in fear expression over time, suggesting that extinction can attenuate fear (mixed ANOVA: F (1,10) = 261.9, P < 0.0001, time factor). **c** Animals exposed to closed-loop stimulation required less extinction sessions to achieve the remission criterion compared to the open-loop group (Mann Whitney test: U = 2, P = 0.0108, two-tailed). **d** The enhancement was also expressed during renewal test since closed-loop neuromodulation induce lower fear expression (Mann Whitney test: U = 0, P = 0.0022, two-tailed). **e** During the

reinstatement test, closed-loop prevents the fear recovery induced by an immediate foot-shock procedure (Mann Whitney test: U = 3, P = 0.0152, two-tailed). * $P < 0.05$, ** $P < 0.01$, *** $P < 0.001$. Bar plots and error bars represent medians and interquartile ranges, individual data points are also displayed. Detailed statistics are shown in Supplementary Data 1. Source data provided as a Source Data file.

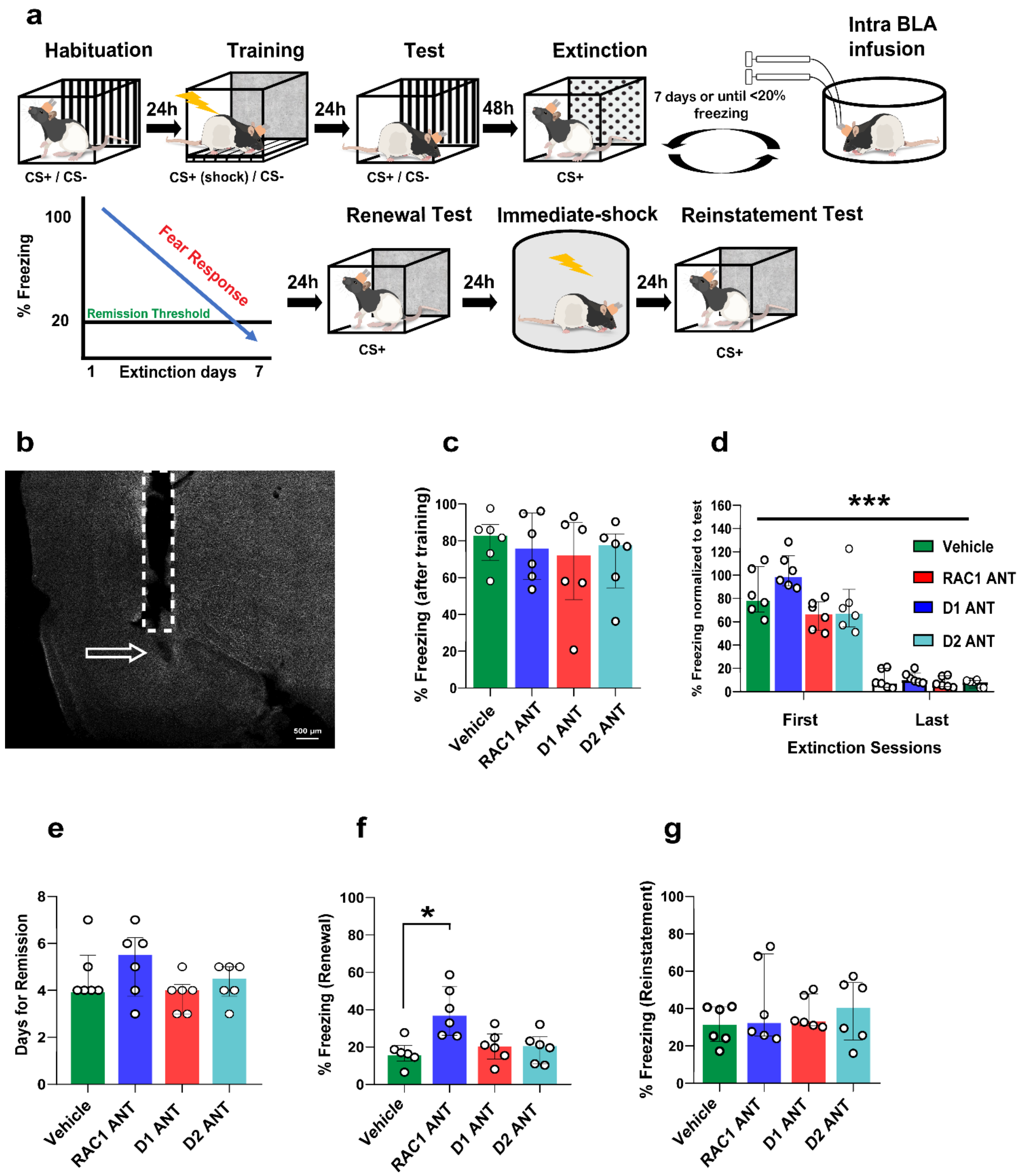

Supplementary Fig. 9. Pharmacological evaluation of NSC2376, SCH23390 and Sulpiride in absence of closed-loop stimulation. a Schematics of the experimental design. **b** Representative histological verification of a cannula placement. The implantation and bilateral placement of the cannula in the BLA were performed according to Fig. 4b. **c** During the test to the CS+, there are no significant differences between the groups, showing that all groups are able to acquire fear

conditioning in a similar fashion (Kruskal-Wallis test: H = 1.060, P = 0.7867). **d** No differences were found in the fear expression between groups during the first 5 CS+ block from first and last extinction day. However, there is a significant decrease in fear expression over time (mixed ANOVA: $F(1,10) = 318.9$, $P < 0.0001$, time factor). **e** The drugs do not have effect itself over the extinction criterion (Kruskal-Wallis test: H = 3.797, P = 0.2843). **f** However, during the renewal, NSC2376 seems to increase freezing behavior only compared to control animals infused with saline (Kruskal-Wallis test: H = 10.86, P = 0.0125). **g** No differences were detected during the reinstatement test (Kruskal-Wallis test: H = 1.173, P = 0.7594). Control (Saline; Vehicle) n = 6; NSC2376 (RAC1 ANT) n = 6; SCH23390 (D1 ANT) n = 6; Sulpiride (D2 ANT) n = 6. * $P < 0.05$, *** $P < 0.001$. Bar plots and error bars represent medians and interquartile ranges, individual data points are also displayed. Detailed statistics are shown in Supplementary Data 1. Source data provided as a Source Data file. Silhouettes on panel **a** are obtained from https://github.com/eackermann/ratpack under MIT License.

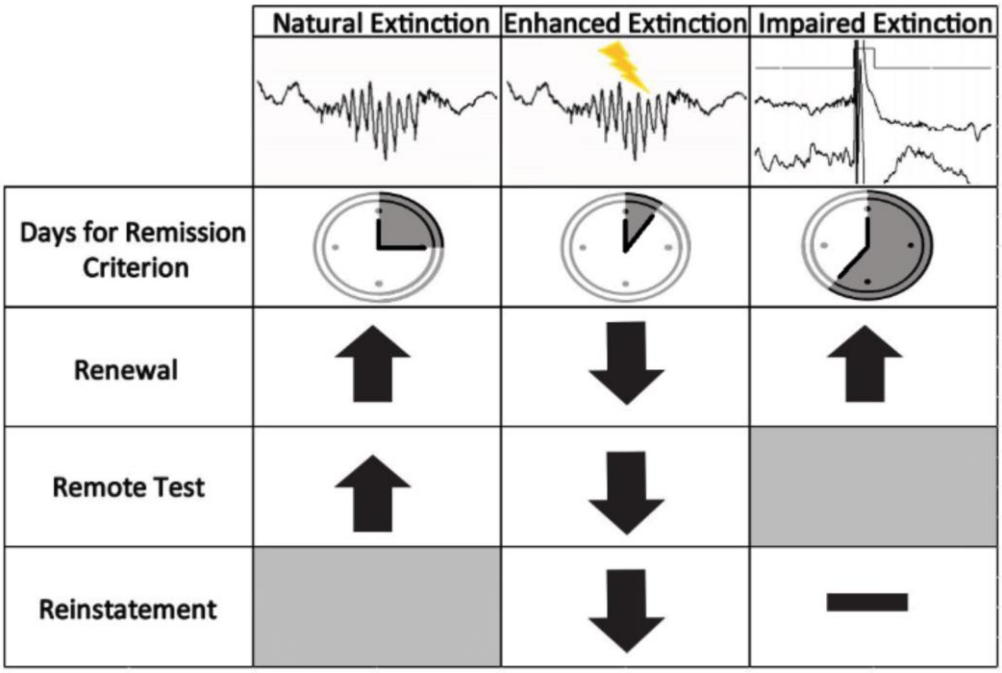

Supplementary Fig. 10. Enhancement of Fear Extinction via Closed-Loop Brain Stimulation. While natural extinction processes (observed in non-stimulated animals) are efficacious in attenuating fear responses, our closed-loop methodology demonstrates superior outcomes in decreasing the number of sessions required to achieve the extinction criterion. Moreover, it is able to maintain lower fear expression during renewal, reinstatement, and remote testing phases. The observed enhancement depends on the medial forebrain bundle stimulation guided by real-time hippocampal sharp wave ripples (SWRs), and dopamine signaling in the basolateral amygdala. SWRs assume a pivotal role during extinction, given that real-time ripple suppression can prolong the extinction process and promote elevated fear expression during the renewal phase.
