# Supplementary material for: Closed-loop brain stimulation augments fear extinction in male rats
Source: Nat Commun. 2023 Jul 5;14:3972. doi: 10.1038/s41467-023-39546-7 (PMC10322911; doi:10.1038/s41467-023-39546-7)
Supplement: Supplementary file 3 — Description of Additional Supplementary Files [file 41467_2023_39546_MOESM3_ESM.pdf]

File name: Supplementary Data 1

Description: Detailed statistical results. The table shows the detailed results and parameters of the descriptive statistics and the statistical tests for each panel of the figures, where applicable, and it also refers to the corresponding conclusions drawn.
