# Supplementary material for: Closed-loop brain stimulation augments fear extinction in male rats
Source: Nat Commun. 2023 Jul 5;14:3972. doi: 10.1038/s41467-023-39546-7 (PMC10322911; doi:10.1038/s41467-023-39546-7)
Supplement: Supplementary file 5 — Reporting Summary [file 41467_2023_39546_MOESM5_ESM.pdf]

nature portfolio

Corresponding author(s): Antal Berényi

Last updated by author(s): 10/05/2023

Reporting Summary

Nature Portfolio wishes to improve the reproducibility of the work that we publish. This form provides structure for consistency and transparency in reporting. For further information on Nature Portfolio policies, see our Editorial Policies and the Editorial Policy Checklist.

Statistics

For all statistical analyses, confirm that the following items are present in the figure legend, table legend, main text, or Methods section.

n/a	Confirmed	
☐	☒	The exact sample size (*n*) for each experimental group/condition, given as a discrete number and unit of measurement
☐	☒	A statement on whether measurements were taken from distinct samples or whether the same sample was measured repeatedly
☐	☒	The statistical test(s) used AND whether they are one- or two-sided<br>*Only common tests should be described solely by name; describe more complex techniques in the Methods section.*
☒	☐	A description of all covariates tested
☐	☒	A description of any assumptions or corrections, such as tests of normality and adjustment for multiple comparisons
☐	☒	A full description of the statistical parameters including central tendency (e.g. means) or other basic estimates (e.g. regression coefficient) AND variation (e.g. standard deviation) or associated estimates of uncertainty (e.g. confidence intervals)
☐	☒	For null hypothesis testing, the test statistic (e.g. *F*, *t*, *r*) with confidence intervals, effect sizes, degrees of freedom and *P* value noted<br>*Give P values as exact values whenever suitable.*
☒	☐	For Bayesian analysis, information on the choice of priors and Markov chain Monte Carlo settings
☒	☐	For hierarchical and complex designs, identification of the appropriate level for tests and full reporting of outcomes
☒	☐	Estimates of effect sizes (e.g. Cohen's *d*, Pearson's *r*), indicating how they were calculated

Our web collection on statistics for biologists contains articles on many of the points above.

Software and code

Policy information about availability of computer code

Data collection	Neuronal signals were recorded using a signal-multiplexed neuronal amplifier Amplipex, KJE1001 (Szeged, Hungary).<br>Online analysis of recorded signals was performed using a programmable digital signal processor RX-8, Tucker-Davis Technologies (Alachua, FL, USA.).<br>Images for histological verification were performed using a Zeiss LSM880 scanning confocal microscope (Carl Zeiss).
Data analysis	Behavioral coding and video tracking were performed using the Solomon software (SOLOMON CODER, (András Péter, Budapest, Hungary) and ANY-Maze (Stoelting, Wood Dale, IL, USA, Version 7.20).<br>A custom made sharp-wave ripple detection algorithm was implemented using the Real-Time Processor Visual Design Studio (RPVDSEX) (Tucker-Davis Technologies; Alachua, FL, USA.).<br>LFP traces were prepared using MATLAB (RRID: SCR_001622; Mathworks, Natick, MA, USA).<br>Images from histological verification were processed using the Carl Zeiss software ZEN Digital Imaging for Light Microscopy (RRID: SCR_013672).<br>Statistical analyses were performed using GraphPad Prism 8 software.<br>For the brain states classifications (SWS/REM), SleepScoreMaster toolbox from Buzcode (https://github.com/buzsakilab/buzcode).<br>Time frequency spectrum was calculated in MATLAB using Multitaper Spectral Estimation from the Chronux Toolbox (http://chronux.org/).

For manuscripts utilizing custom algorithms or software that are central to the research but not yet described in published literature, software must be made available to editors and reviewers. We strongly encourage code deposition in a community repository (e.g. GitHub). See the Nature Portfolio guidelines for submitting code & software for further information.

Data

Policy information about availability of data

All manuscripts must include a data availability statement. This statement should provide the following information, where applicable:

- Accession codes, unique identifiers, or web links for publicly available datasets
- A description of any restrictions on data availability
- For clinical datasets or third party data, please ensure that the statement adheres to our policy

The data generated in this study (in the main manuscript and in the Supplementary Information) are provided in the Source Data file and Supplementary Data, or from the corresponding author upon reasonable request. Source data are provided with this paper. All custom code is freely available from the corresponding author on reasonable request.

Human research participants

Policy information about studies involving human research participants and Sex and Gender in Research.

Reporting on sex and gender	N/A
Population characteristics	N/A
Recruitment	N/A
Ethics oversight	N/A

Note that full information on the approval of the study protocol must also be provided in the manuscript.

Field-specific reporting

Please select the one below that is the best fit for your research. If you are not sure, read the appropriate sections before making your selection.

☒ Life sciences ☐ Behavioural & social sciences ☐ Ecological, evolutionary & environmental sciences

For a reference copy of the document with all sections, see nature.com/documents/nr-reporting-summary-flat.pdf

Life sciences study design

All studies must disclose on these points even when the disclosure is negative.

Sample size	No statistical methods were used to pre-determine sample size, but our sample sizes are similar to those reported in previous publications with similar experimental design (e.g. Yang Y., Nat Neurosci. 2016).
Data exclusions	Rats with poor fear expression after fear conditioning were excluded.<br>During local pharmacological manipulation, animals with cannuli missing basolateral amygdala were excluded.
Replication	The main findings from our study on extinction enhancement using closed-loop neuromodulation are illustrated in Figure 1. These findings were successfully replicated in Figure 4 and the Supplementary Figure 8, with an independent sample of animals used in all cases.
Randomization	For all experiments, rats were randomly allocated into each experimental group. In case of parallel behavioral task, the order of sessions were randomized across the experiment.
Blinding	During behavioral coding, the experienced observer evaluating the results was blinded to the experimental group. The electrophysiological analyses were conducted by researchers who were different from those involved in behavior recording and blinded to the experimental conditions.

Reporting for specific materials, systems and methods

We require information from authors about some types of materials, experimental systems and methods used in many studies. Here, indicate whether each material, system or method listed is relevant to your study. If you are not sure if a list item applies to your research, read the appropriate section before selecting a response.

Materials & experimental systems

n/a	Involved in the study
☒	☐ Antibodies
☒	☐ Eukaryotic cell lines
☒	☐ Palaeontology and archaeology
☐	☒ Animals and other organisms
☒	☐ Clinical data
☒	☐ Dual use research of concern

Methods

n/a	Involved in the study
☒	☐ ChIP-seq
☒	☐ Flow cytometry
☒	☐ MRI-based neuroimaging

Animals and other research organisms

Policy information about studies involving animals; ARRIVE guidelines recommended for reporting animal research, and Sex and Gender in Research

Laboratory animals	Rats (120 adult male Long-Evans, 300-450 g, 3-6 months old) were kept in a 12-hour light/ dark cycle. Please see details in the Methods section.
Wild animals	The study did not involve wild animals
Reporting on sex	Only male Long-Evans rats were used in this study.
Field-collected samples	The study did not involve any field-collected samples
Ethics oversight	All experiments were performed in accordance with the European Union guidelines (2003/65/CE) and the National Institutes of Health Guidelines for the Care and Use of Animals for Experimental Procedures. The experimental protocols were approved by the Ethical Committee for Animal Research at the Albert Szent-Györgyi Medical and Pharmaceutical Center of the University of Szeged (XIV/218/2016 and XIV/824/2021).

Note that full information on the approval of the study protocol must also be provided in the manuscript.
